# Efficacy and safety of remimazolam tosylate for sedation in ICU patients: A multicenter, randomized, phase 2 study

**DOI:** 10.1016/j.jointm.2026.01.009

**Published:** 2026-03-28

**Authors:** Ning Liu, Fenghui Lin, Jianli Wen, Xiangyou Yu, Fachun Zhou, Zhanbiao Yu, Mei Yang, Li Yu, Ailian Lv, Yun Sun, Feng Shen, Bin He, Zhenjie Hu, Tengrui Yin, Xi Li, Huixin Zhao, Jianfeng Wu, Chuanxi Chen, Yao Nie, Lingyun Zuo, Xiangdong Guan, Minying Chen

**Affiliations:** 1 Department of Critical Care Medicine, The First Affiliated Hospital, Sun Yat-sen University, Guangzhou, Guangdong, China; 2 Department of Intensive Care Unit, Fuzhou University Affiliated Provincial Hospital, Fuzhou, Fujian, China; 3 Department of Intensive Care Unit, The First People’s Hospital of Zunyi City, Zunyi, Guizhou, China; 4 Department of Critical Care Medicine, The First Affiliated Hospital of Xinjiang Medical University, Urumqi, Xinjiang, China; 5 Department of Intensive Care Unit, The First Affiliated Hospital of Chongqing Medical University, Chongqing, China; 6 Department of Intensive Care Unit, Affiliated Hospital of Hebei University, Baoding, Hebei, China; 7 Department of Intensive Care Unit, Qujing Central Hospital of Yunnan Province, Qujing, Yunnan, China; 8 Department of Intensive Care Unit, Wuhan Central Hospital, Wuhan, Hubei, China; 9 Department of Critical Care Medicine, The First Hospital of Changsha City, Changsha, Hunan, China; 10 Department of Critical Care Medicine, The Second Hospital of Anhui Medical University, Hefei, Anhui, China; 11 Department of Critical Care Medicine, The Affiliated Hospital of Guizhou Medical University, Guiyang, Guizhou, China; 12 Department of Intensive Care Unit, Shanghai Chest Hospital, Shanghai, China; 13 Department of Intensive Care Unit, The Fourth Hospital of Hebei Medical University, Shijiazhuang, Hebei, China; 14 Department of Clinical Pharmacology, Jiangsu Hengrui Pharmaceuticals Co., Ltd., Shanghai, China; 15 Department of Biometrics, Jiangsu Hengrui Pharmaceuticals Co., Ltd., Shanghai, China; 16 Department of Central Neuromedicine, Jiangsu Hengrui Pharmaceuticals Co., Ltd., Shanghai, China

**Keywords:** Remimazolam tosylate, Intensive care unit, Sedation, Mechanical ventilation

## Abstract

**Background:**

Effective sedation in intensive care unit (ICU) patients is critical for minimizing agitation, ensuring safety, and facilitating mechanical ventilation. Remimazolam tosylate, a novel benzodiazepine with rapid onset and short duration, metabolized by tissue esterases, represents a promising agent for ICU sedation. This study aimed to evaluate the efficacy and safety of remimazolam tosylate in short-term sedation for ICU patients.

**Methods:**

This was a multicenter, randomized, dose-finding, phase 2 study. From October 12, 2022 through April 19, 2023, ICU patients requiring mechanical ventilation and sedation for ≥6 h were randomized (1:1) into two groups: low adjustment rate (LAR) group received intravenous remimazolam tosylate with a maintenance rate adjustment of 0.1 mg/kg/h, and high adjustment rate (HAR) group with 0.2 mg/(kg·h). Both groups were initiated at an infusion rate of 0.2 mg/(kg·h) with the same loading dose (0.08 mg/kg) and maintenance range (0–2.0 mg/(kg·h)). The primary outcome was sedation success rate, defined as the percentage of time maintaining targeted sedation for ≥70% of the drug administration period without rescue sedation.

**Results:**

A total of 59 patients completed the study (LAR, *n*=30; HAR, *n*=29). Within the limited sample, all patients achieved sedation success. The median percentage of time within the target sedation maintenance relative to remimazolam administration duration was 97.9% (IQR: 93.8%–99.7%) for LAR group and 98.1% (IQR: 93.6%–99.8%) for HAR group (*P*=0.7617). No patients required rescue sedation with propofol, and only one patient (3.3%) in LAR group required rescue analgesia with sufentanil. Most treatment-emergent adverse events (TEAEs) were mild or moderate, with hypotension (18.6%), hypertension (18.6%), and anemia (16.9%) being the most common. One serious TEAE (3.4%, disease progression) and one death were reported in HAR group, both were assessed as unrelated to remimazolam tosylate.

**Conclusions:**

Remimazolam tosylate demonstrated high efficacy with manageable safety profile for short-term sedation in mechanically ventilated ICU patients at both 0.1 mg/(kg·h) and 0.2 mg/(kg·h) adjustment rates.

**Trial registration:** ClinicalTrials.gov, NCT05152303

## Introduction

Sedation is clinically indicated for the majority of mechanically ventilated patients in the intensive care unit (ICU) to ensure comfort, safety, and treatment compliance.^[^[1], [2], [3], [4]^]^ Current sedation protocols predominantly rely on midazolam, propofol, and dexmedetomidine. However, concerns have been raised regarding the use of these commonly used ICU sedatives due to the recognized problems such as propofol infusion syndrome, long and unpredictable wake-up times, delirium, respiratory depression and life-threatening bradycardia,^[^^3^^,^[5], [6], [7]^]^ underscoring the need for safer alternatives.

Remimazolam is a novel ultra-short-acting benzodiazepine that is metabolized rapidly to an inactive carboxylic acid metabolite by nonspecific tissue esterase, and therefore, it has a rapid and a predictable onset and offset profile.^[^^8^^,^^9^^]^ Evidence from procedural sedation in high-risk (American Society of Anesthesiologists [ASA] Ⅲ/Ⅳ) patients undergoing colonoscopy demonstrates that remimazolam achieves therapeutic efficacy comparable to propofol while offering superior hemodynamic stability, reduced respiratory depression, and potentially fewer neurocognitive complications.^[^^10^^]^ Clinical studies for mechanically ventilated patients in ICU support the efficacy and safety of remimazolam besylate for sedation, with hemodynamic profiles comparable to those of propofol.^[^^11^^,^^12^^]^ The tosylate formulation (remimazolam tosylate [HR7056]) was developed with the aim of improving physicochemical properties, including enhanced optical purity and stability. Remimazolam tosylate has been approved in China for procedural sedation in non-intubated patients and for induction and maintenance of general anesthesia.^[^^13^^]^ Comparative studies have confirmed equivalent anesthetic efficacy and safety profiles between remimazolam tosylate and remimazolam besylate in clinical settings such as daytime hysteroscopic surgery.^[^^14^^]^

While remimazolam’s pharmacological advantages are well-established in procedural sedation and general anesthesia, its role in critical care medicine remains under characterized. Particularly lacking are robust clinical data evaluating its application in ICU settings where complex pathophysiology and concurrent organ dysfunction may significantly alter its pharmacodynamic profile. Herein, we conducted a multicenter, randomized trial to evaluate the efficacy and safety of remimazolam tosylate for short-term sedation in mechanically ventilated ICU patients.

## Methods

### Study design and patients

This was a multicenter, randomized, dose-finding, phase 2 study (ClinicalTrials.gov, NCT05152303) of remimazolam tosylate for short-term sedation in mechanically ventilated ICU patients conducted across 13 sites in China (Supplementary Table S1). The protocol and all amendments were approved by the Ethics Committee of each participating site and has been previously published.^[^^15^^]^ The study was conducted in accordance with the Declaration of Helsinki and Good Clinical Practice guidelines. Written informed consent was required for all patients or their guardians. The first patient was enrolled on October 12, 2022, and the final date of follow-up was April 19, 2023.

Patients eligible for randomization aged ≥18 to ≤80 years with a body mass index (BMI) >18 kg/m^2^ to <30 kg/m^2^ who were admitted to the ICU and were expected to require continuous mechanical ventilation and sedation for at least 6 h, with a target Richmond Agitation-Sedation Scale (RASS) of −2 to +1. Key exclusion criteria included requiring deep sedation for organ-protection; requiring neuromuscular blocking agents during sedation maintenance (excluding intubation procedures); history of severe cardiovascular, cerebrovascular, or neuropsychiatric disorders; clinically significant laboratory abnormalities; abnormal blood pressure or heart rate; pregnancy or nursing. Full inclusion and exclusion criteria are provided in Supplementary Table S2.

### Randomization and blinding

The study consisted of *a* ≤ 7-day screening period, a 6- to 24-h treatment period, a 24-h follow-up period (Supplementary Figure S1). Eligible patients were randomly assigned (1:1) to the low adjustment rate (LAR) group and high adjustment rate (HAR) group to receive remimazolam tosylate intravenously at different infusion adjustment rates. The randomization was performed by an independent statistician using SAS software. Allocation concealment was maintained using a centralized electronic randomization system that assigned treatments only after patient enrollment was completed. Due to protocol-mandated variations in infusion rates that inherently compromised blinding integrity, the patients and evaluating investigator were blinded to treatment assignment. To maintain blinding integrity, a strict functional separation was maintained throughout the study. The infusion system was physically concealed during drug administration. All treatment interventions and dose adjustments were executed exclusively by the unblinded clinicians, who had no role in patient assessments. The blinded investigators performed all assessments and determined the need for dosage modifications based on clinical evaluations, but was excluded from actual titration procedures. Multiple investigators from both groups were available to ensure continuous protocol adherence. Patients remained unaware of their treatment assignment throughout the study period.

### Study drug administration

Sedation and analgesia prior to initiation of remimazolam tosylate are detailed in the Supplementary Methods. Remimazolam tosylate was administered intravenously at a loading dose of 0.08 mg/kg and an initial infusion rate of 0.2 mg/(kg·h) for both groups and adjusted at 0.1 mg/(kg·h) in the LAR group, at 0.2 mg/(kg·h) in the HAR group, within a range of 0–2.0 mg/(kg·h), to maintain target sedation range (RASS −2 to +1).

Following any invasive or stimulating procedure (e.g., airway suctioning, intramuscular injections, tracheoscopy, tracheostomy, and thoracic paracentesis), a single bolus of 0.08 mg/kg could be given over 1 to 2 min if RASS >+1. Only one additional bolus is permitted per procedure. If the RASS score remains >+1 3 min after additional bolus, the maintenance infusion rate of remimazolam tosylate was to be increased in stepwise increments to achieve target sedation range.

If a RASS score of ≤ −3 was recorded for ≥15 min during maintenance at the minimum infusion rate, the administration of remimazolam tosylate was to be temporarily discontinued. The infusion was to be restarted at an initial rate of 0.2 mg/(kg·h) only after the RASS score had recovered to ≥0.

If the maximum permitted maintenance infusion rate persisted for ≥15 min without achieving a sedation level of RASS ≤+1, rescue sedation with propofol was administered intravenously (0.3–0.5 mg/kg, maximum of 5 doses at ≥3-min intervals) while the remimazolam infusion was maintained. If the target RASS score of ≤+1 was not achieved after five doses of intravenous propofol, remimazolam administration was discontinued. Otherwise, maintenance infusion of remimazolam tosylate continued until sedation was no longer required as determined by the investigator, the 24-hour duration limit was reached, or safety concerns occurred (e.g. severe respiratory, neurological, or circulatory complications).

For analgesia, continuous fentanyl citrate infusion was initiated at 0.5 µg/(kg·h) for all patients, starting 5 min before the initiation of remimazolam tosylate and maintained throughout its administration. The infusion rate of fentanyl citrate was titrated within 0.5–1.0 µg/(kg·h). Incremental adjustments of 0.1 µg/(kg·h) were permitted only if the Critical Care Pain Observation Tool (CPOT) score exceeded 2, with real-time CPOT monitoring mandatory to guide dose optimization. If CPOT remained >2 at the maximum infusion rate, rescue analgesia with single intravenous bolus of sufentanil at 0.5 µg/kg was given.

### Sedation assessment and monitoring

After successful randomization and propofol discontinuation, RASS was assessed every (15±5) minutes or as needed by investigators. Baseline RASS was measured within 10 min before remimazolam tosylate administration. Post-initiation, RASS evaluations occurred at 1, 2, 3, 5, 10, and 30 min; then 1, 2, 4, 6, 8, 12, 16, 20, and 24 h. Immediate RASS assessment was performed upon infusion discontinuation; subsequent scheduled assessments were discontinued.

The timing of RASS assessments relative to interventions was protocolized according to the nature of the clinical event. For scheduled procedures, blinded investigators performed assessments within 5 min before the procedure. For unscheduled events (e.g., sudden agitation), unblinded clinicians immediately contacted blinded investigators for assessment. Post-procedural RASS evaluations were required within 5 min after any infusion rate adjustment, additional dose administration or rescue sedation. All formal RASS assessments were conducted exclusively by blinded investigators prior to subsequent interventions, with investigators retaining discretion to perform additional RASS evaluations based on clinical need.

CPOT assessments were performed concurrently to guide analgesia titration, as detailed in Supplementary Methods. Safety assessments included incidence and severity of adverse events (AEs), vital signs, physical examinations, laboratory tests, and 12-lead electrocardiogram were performed up to 24 hours after discontinuation of remimazolam tosylate.

### Outcomes

The primary outcomes was the proportion of patients achieving sedation success, defined as maintaining a target sedation level (RASS −2 to +1) for ≥70% of the total drug administration period without requiring rescue sedatives. RASS assessments served both as triggers for treatment intervention and as the basis for outcomes calculation. Secondary outcomes included percentage of time within target sedation range, proportion of patients receiving rescue sedation/analgesia, mean dose of sufentanil as rescue analgesia, number of infusion rate adjustments; time to full wakefulness (defined as time to recovery to RASS ≥0 after discontinuation of remimazolam tosylate), duration of mechanical ventilation and nursing score.

### Pharmacokinetics assessments

Blood samples were collected to determine the plasma concentration of remimazolam tosylate (HR7056) and its metabolite (HR7054) at 10 minutes and 2 hours after infusion initiation, at the end of infusion and at 1, 2 and 4 h after discontinuation of remimazolam tosylate. Pharmacokinetic (PK) parameters were calculated, including maximum plasma concentration (C_max_), area under the concentration-time curve from zero to the last measurable concentration (AUC_0-t_), elimination half-life (t_1/2_), apparent clearance (CL/F), and apparent volume of distribution (V_z_/F).

### Statistical analysis

This was an exploratory phase 2 study, the sample size was not based on a formal power calculation but was consistent with previous phase 2 clinical studies of remimazolam tosylate in other indications and similar drugs in ICU sedation.^[^^16^^,^^17^^]^ Sixty patients were planned to be enrolled into the two groups of the study with a 1:1 ratio (30 per group). No formal statistical hypothesis testing was planned. Efficacy analyses were performed in the full analysis set (FAS), which included all randomized patients who received at least one dose of the study treatment. The primary outcome was based on FAS and was also analysed in per protocol analysis set (PPS), included patients who received at least one dose of the study treatment without major protocol deviations. Safety was analysed in all patients who received at least one dose of the study treatment and at least one post-administration safety assessment.

Continuous variables are summarized as mean ± standard deviation or median (interquartile range [IQR]), as appropriate. Categorical variables are presented as number (percentage). Time-to-event variables are expressed as median time with 95% confidence interval (CI) and compared using the log-rank test. For the sedation success rate, 95% CIs are calculated using the Clopper-Pearson exact method. Comparisons between LAR group *vs.* HAR group were performed using the Wilcoxon rank-sum test for percentage of time within target sedation level, total number of infusion rate adjustment, duration of mechanical ventilation and nursing score. Pharmacokinetic parameters were estimated through non-compartmental analysis (NCA) using SAS (version 9.4).

## Results

### Patients and treatment

**Figure 1:**
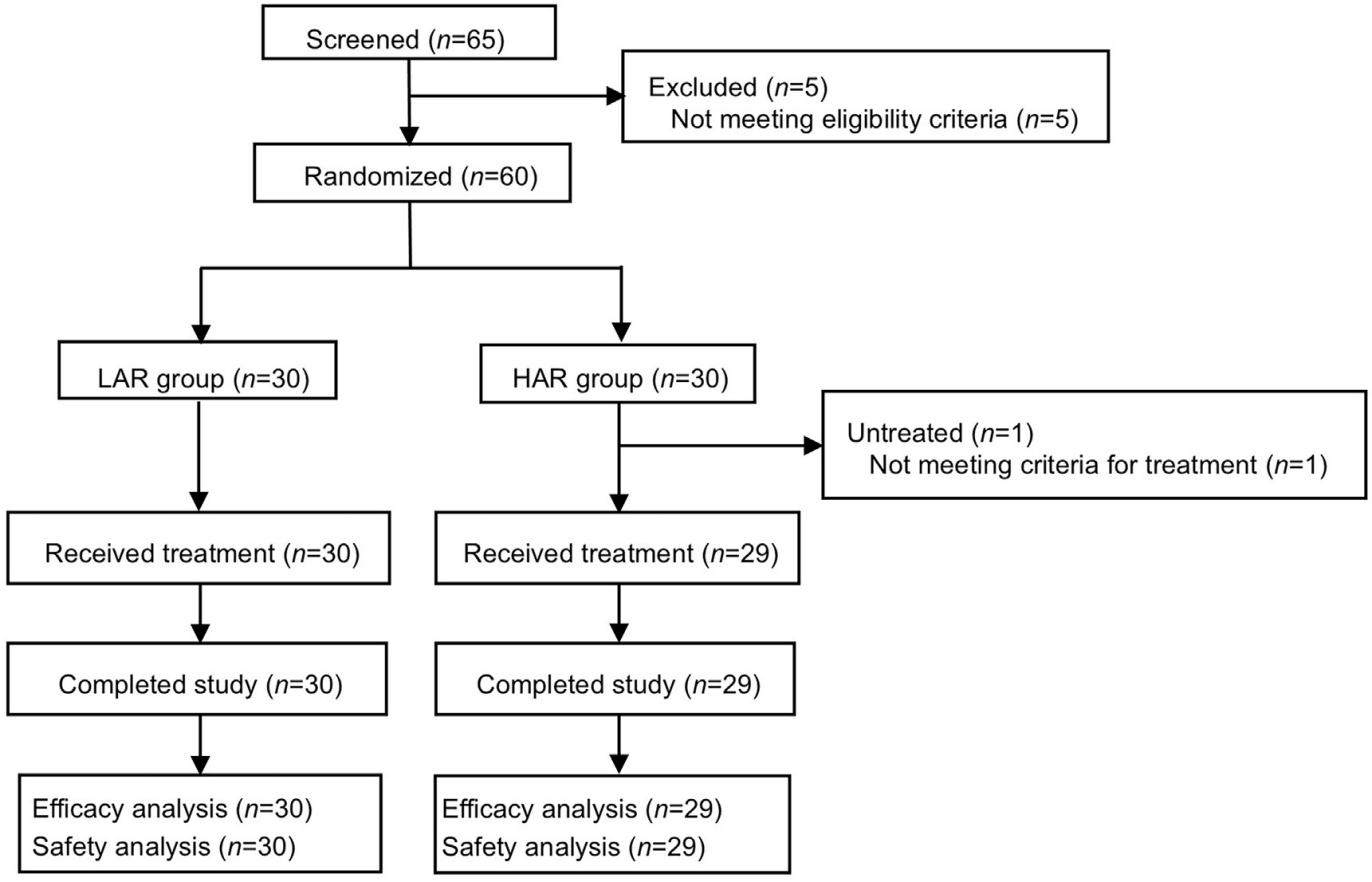
Trial profile of the study. LAR: Low adjustment group; HAR: High adjustment group.

From October 12, 2022 to April 19, 2023, 65 patients were screened, and 60 patients were enrolled and randomly assigned to the LAR group (*n*=30) and HAR group (*n*=30). One patient in HAR group withdrew from the study due to failure to achieve RASS ≥2 at 60 min after propofol discontinuation. A total of 59 patients received assigned treatment of remimazolam tosylate and completed study (Figure 1).

Patient demographics and baseline characteristics were generally balanced between the two groups (Table 1). The median age of the patients was 66.5 (IQR: 50.0–72.0) years in LAR group and 63.0 (IQR: 55.0–70.0) years in HAR group; the median RASS score at baseline was 2 (IQR: 2–2) in both groups. All patients were admitted to ICU due to surgical reasons.

**Table 1:** Demographics and baseline characteristics**.**

Characteristics	LAR group (*n*=30)	HAR group (*n*=29)
Age (years)	66.5 (50.0–72.0)	63.0 (55.0–70.0)
Sex		
Male	20 (66.7)	20 (69.0)
Female	10 (33.3)	9 (31.0)
Weight (kg)	61.7 ± 11.6	63.3 ± 11.8
BMI (kg/m^2)^	22.6 (20.3–25.4)	22.6 (20.8–25.8)
Post-surgery admission		
Yes	30 (100.0)	29 (100.0)
No	0	0
Heart rate (beats/min)	92.0 (80.0–101.0)	84.0 (69.0–100.0)
RASS score	2 (2–2)	2 (2–2)
SOFA score	2 (2–3)	2 (1–3)
Muscle tone score	3 (3–3)	3 (3–3)

Data are presented as *n* (%), mean ± standard deviation or median (interquartile range).

BMI: Body mass index; HAR: High adjustment rate; LAR: Low adjustment rate; RASS: Richmond Agitation-Sedation Scale; SOFA: Sequential Organ Failure Assessment.

### Efficacy

**Figure 2:**
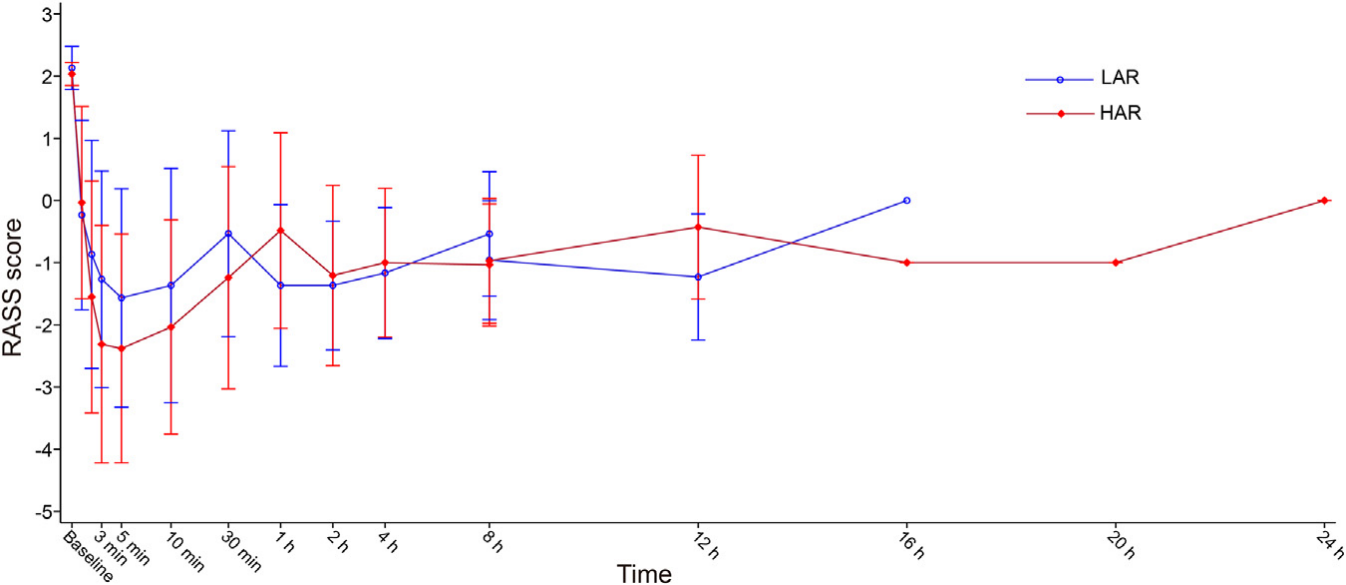
RASS scores during remimazolam tosylate administration. RASS scores during administration of the trial drug, shown as mean ± standard deviation. HAR: High adjustment rate; LAR: Low adjustment rate; RASS: Richmond Agitation-Sedation Scale.

Both groups achieved 100% successful sedation. The median percentage of time within target sedation range was comparable between two groups (LAR, 97.9% [IQR: 93.8%−99.7%; HAR, 98.1% [IQR: 93.6%−99.8%; *P*=0.7617), with an overall median of 98.1% (IQR: 93.6%–99.8%) across all patients. The RASS scores over the dosing period are shown in Figure 2. Other efficacy outcomes were also comparable between two groups (Table 2), including number of infusion rate adjustments (2.0 [IQR: 0–4.0] *vs.* 1.0 [IQR: 0–4.0], *P*=0.4572). Further analysis of titration direction showed that the number of upward adjustments was comparable between the LAR and HAR groups (0.5 [IQR: 0–3.0] *vs.* 0 [IQR: 0–1.0], *P=*0.6638), as was the number of downward adjustments (1.0 [IQR: 0–2.0] *vs.* 0 [IQR: 0–2.0], *P=*0.7026) (Supplementary Table S3). Nursing scores (IQR: 5.5 [5.0–7.0] *vs.* 5.0 [IQR: 5.0–7.0], *P=*0.8823) and duration of mechanical ventilation (13.1 [IQR: 8.9–14.9] h *vs.* 12.7 [IQR: 10.3–14.3] h, *P=*0.9516) (Supplementary Figure S2). Most patients recovered to full wakefulness immediately after discontinuation of remimazolam tosylate, with the time was (8.6±17.5) minutes in LAR group and (4.4±9.4) minutes in HAR group (Supplementary Figure S3), and an overall wakefulness time of (6.6±14.3) minutes across all patients. One patient in LAR group received rescue analgesia of sufentanil, no patients received rescue sedatives in the study. Additionally, target sedation maintained for 75%–99% of the total infusion duration, with 18/30 (60.0%) patients in the LAR group and 21/29 (72.4%) patients in the HAR group requiring no rate adjustments within the first 15 min.

**Table 2:** Primary and secondary outcomes of the study.

Outcomes	LAR group (*n*=30)	HAR group (*n*=29)	*P* value
Primary outcome			
Sedation success	30 (100)	29 (100)	–
95% CI*	88.1–100.0	86.3–100.0	
Secondary outcomes			
Percentage of time within target sedation level (%)	97.9 (93.8–99.7)	98.1 (93.6–99.8)	0.7617
Receiving rescue sedation medication	0	0	–
Receiving rescue analgesia medication	1(3.3)	0	
Additional dose of sufentanil (µg)†	33.5	0	–
Total number of infusion rate adjustment	2.0 (0–4.0)	1.0 (0–4.0)	0.4572
Time to full wakefulness (min)‡	0 (0–6.0)	0 ( NA–NA)	
Duration of mechanical ventilation (h)	13.1 (8.9–14.9)	12.7 (10.3–14.3)	0.9516
Nursing score	5.5 (5.0–7.0)	5.0 (5.0–7.0)	0.8823

Data are presented as *n* (%), median (95% CI) or median (interquartile range).

CI: Confidence interval; HAR: High adjustment rate; LAR: Low adjustment rate; NA: Not applicable.

⁎ For the sedation success rate, 95% CI were calculated using the Clopper-Pearson exact method.

† Only one patient in this group received sufentanil as rescue analgesia; data shown are for this single case.

‡ The median time to full wakefulness was calculated using the Kaplan-Meier method, 95% CI was calculated using the Brookmeyer-Crowley method, the comparison was performed using the log-rank test. The 95% CI in the HAR group could not be estimated using the Brookmeyer – Crowley method because all events occurred at time zero with no censoring, resulting in a degenerate survival curve. CI is therefore reported as (NA, NA).

### Safety and tolerability

All 59 treated patients were included in the safety analysis. Treatment-emergent AEs (TEAEs) occurred in 28 (93.3%) patients in the LAR group and 25 (86.2%) patients in the HAR group, majority of which were mild or moderate in severity. Two patients in HAR group reported severe TEAEs. Treatment-related AEs (TRAEs) occurred in 13 (43.3%) patients in the LAR group and 6 (20.7%) patients in the HAR group, the majority were mild in severity. One serious TEAE of disease progression (resulting in death) occurred in a 77-year-old male patient in HAR group, and was assessed as unrelated to remimazolam tosylate treatment. No other TEAEs led to treatment discontinuation.

TEAEs of LAR and HAR group were presented in Table 3. The most common (≥10.0%) TEAEs following remimazolam tosylate treatment for all patients in both groups were hypotension (18.6%), anemia (16.9%), hypertension (18.6%), pyrexia (11.9%), increased white blood cell count (11.9%), hypoproteinemia (11.9%) and respiratory alkalosis (10.2%). The most common TRAEs included hypotension (15.3%), respiratory depression (5.1%) and bradycardia (3.3%).

**Table 3:** Safety summary and common TEAEs.

Events	Overall (*n*=59)	LAR group (*n*=30)	HAR group (*n*=29)
Any TEAEs	53 (89.8)	28 (93.3)	25 (86.2)
Mild	31 (52.5)	16 (53.3)	15 (51.7)
Moderate	20 (33.9)	12 (40.0)	8 (27.6)
Severe	2 (3.4)	0	2 (6.9)
Any TRAEs	19 (32.2)	13 (43.3)	6 (20.7)
Mild	15 (25.4)	10 (33.3)	5 (17.2)
Moderate	4 (6.8)	3 (10.0)	1 (3.4)
Severe	0	0	0
TE-SAEs	1 (1.7)	0	1 (3.4)*
TR-SAEs	0	0	0
TEAEs leading to death	1 (1.7)	0	1 (3.4)*
TRAEs leading to death	0	0	0
Common TEAEs (≥10.0% in either group)			
Hypotension	11 (18.6)	7 (23.3)	4 (13.8)
Hypertension	11 (18.6)	7 (23.3)	4 (13.8)
Anemia	10 (16.9)	7 (23.3)	3 (10.3)
Pyrexia	7 (11.9)	5 (16.7)	2 (6.9)
White blood cell count increased	7 (11.9)	5 (16.7)	2 (6.9)
Hypoproteinemia	7 (11.9)	3 (10.0)	4 (13.8)
Respiratory alkalosis	6 (10.2)	2 (6.7)	4 (13.8)
Blood pressure decreased	5 (8.5)	4 (13.3)	1 (3.4)
Blood pressure increased	5 (8.5)	4 (13.3)	1 (3.4)
Neutrophil count increased	5 (8.5)	3 (10.0)	2 (6.9)
Aspartate aminotransferase increased	4 (6.8)	0	4 (13.8)
Inflammation	4 (6.8)	3 (10.0)	1 (3.4)
Pneumonia	3 (5.1)	3 (10.0)	0
Hypophosphatemia	3 (5.1)	0	3 (10.3)
Blood glucose increased	3 (5.1)	0	3 (10.3)

Data are presented as *n* (%).

HAR: High adjustment rate; LAR: Low adjustment rate; TEAE: Treatment-emergent adverse event; TRAE: Treatment-related adverse event; TE-SAE: Treatment-emergent serious adverse event; TR-SAE: Treatment-related serious adverse event.

⁎ One patient died due to SAE of disease progression leading to multi-organ failure. The deceased was a 77-year-old male. The event was assessed by both the investigator and sponsor as unrelated to remimazolam.

### Treatment exposure

Treatment exposure characteristics were presented in Supplementary Table S4. The duration of remimazolam tosylate administration (time from loading to discontinuation) was (624.1±196.6) minutes in LAR group and (664.7±267.3) minutes in HAR group, with maximum durations of 1113.7 min and 1437.0 min, respectively. Patients received a loading dose of (5.0±0.9) mg (LAR: [4.9±0.9] mg; HAR: [5.1±0.9] mg). The mean total dose of remimazolam tosylate was (138.5 ± 101.7) mg in LAR group and (143.7±88.1) mg in HAR group. Three patients (5.1%) required additional dose of remimazolam tosylate (LAR: *n*=1, 3.3%; HAR: *n*=2, 6.9%), with the supplemental dose of 4.1 (LAR), 4.2 and 5.6 (HAR) mg, respectively.

Fentanyl was administered for a duration of (10.6±4.1) hours (LAR: [10.4±3.3] hours; HAR: [10.8±4.8] hours). The mean total dose of fentanyl was (380.0 ± 203.5) µg in LAR group and (416.3±273.5) µg in HAR group. Thirty-four (57.6%) patients underwent invasive or stimulating procedures and received additional doses of fentanyl ( LAR: *n*=18, 60.0%; HAR: *n*=16, 55.2%).

### Pharmacokinetics

**Figure 3:**
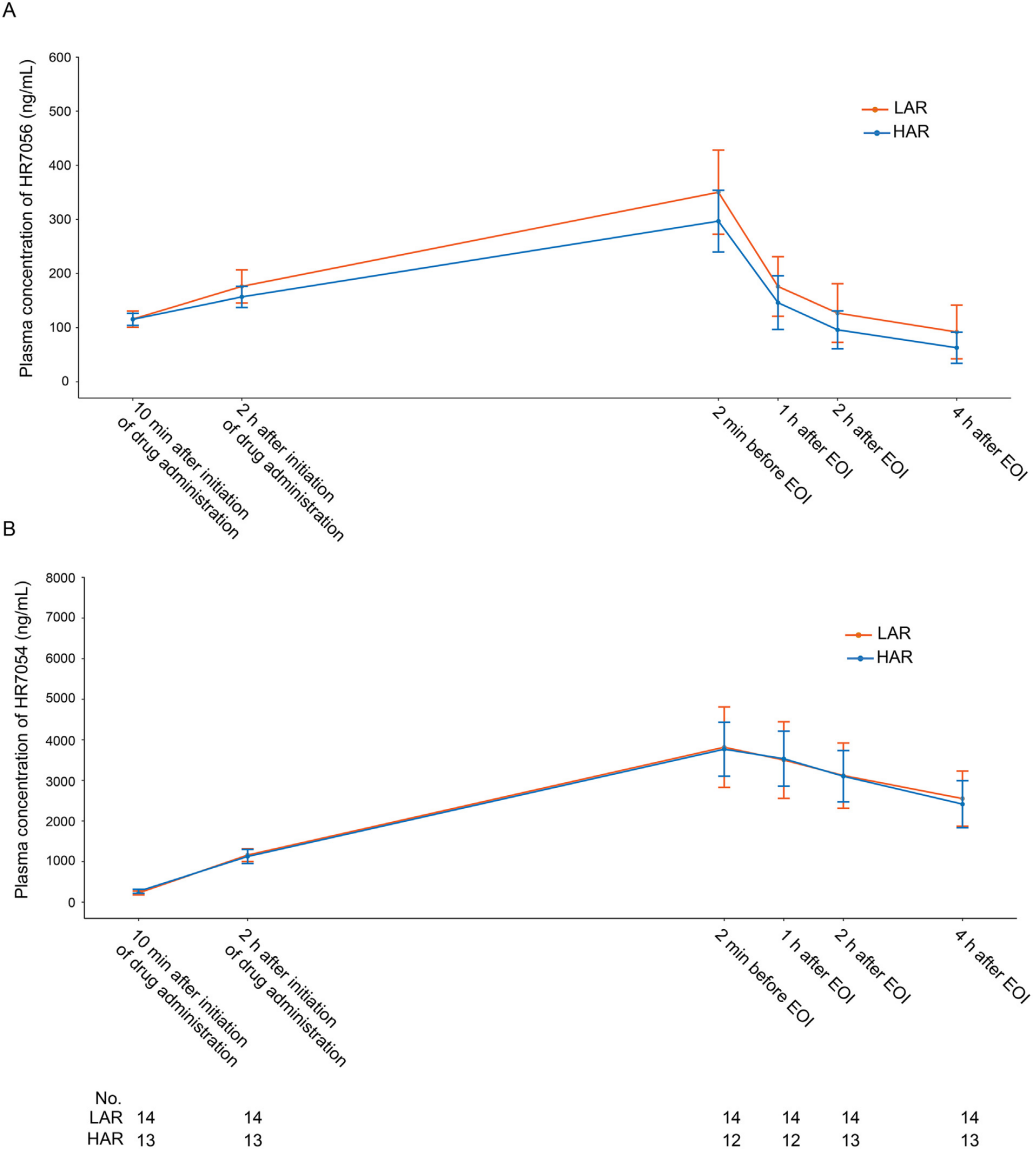
Plasma concentration-time curve of remimazolam tosyate after treatment discontinuation**.** Plasma concentration-time curve of remimazolam tosylate (A) and its metabolite HR7054 (B), shown as mean ± standard error. EOI: End of infusion; HAR: High adjustment rate; LAR: Low adjustment rate.

Twenty-six remimazolam tosylate-treated patients (14 in LAR group and 12 in HAR group) were included in the PK analysis. The mean duration of remimazolam infusion was 11.1 h at a mean rate of 0.21 mg/(kg·h). At 2 h post-initiation and at discontinuation, the mean infusion rates were 0.18 mg/(kg·h) and 0.23 mg/(kg·h), respectively, with mean plasma concentrations of 167 ng/mL and 326 ng/mL. The plasma concentration-time profile of remimazolam tosylate is presented in Figure 3, and PK parameters are summarized in Supplementary Table S5. The plasma concentration of HR7056 decreased rapidly after discontinuation of remimazolam tosylate with a t_1/2_ of 1.65 h.

## Discussion

This multicenter, randomized phase 2 study represents the evaluation of remimazolam tosylate, a rapidly metabolized benzodiazepine, for short-term (<24 h) sedation in mechanically ventilated ICU patients. With two maintenance rate adjustment amplitudes (0.1 mg/(kg·h) and 0.2 mg/(kg·h)) across 59 patients, this study assessed efficacy, safety and PK of remimazolam tosylate for short-term sedation.

Despite current ICU sedation guidelines recommending propofol over midazolam for short-term sedation due to its association with faster recovery times,^[^^18^^]^ multiple studies have demonstrated that remimazolam outperforms propofol in maintaining hemodynamic stability, rendering it a preferable choice for critically ill patients with hemodynamic compromise.^[^^17^^,^^19^^,^^20^^]^ In long-term (>24 h) sedation, previous study reported a median percentage of time within the target RASS range without requiring rescue sedation of 73.2% (IQR: 41.5%–97.3%) for remimazolam besylate and 82.8% (IQR: 65.6%–100%) for propofol.^[^^11^^]^ In this study, remimazolam tosylate achieved a median of 98.1% (IQR: 93.6%–99.8%) during short-term (6–24 h) sedation. Chen et al.^[^^17^^]^ reported that a loading dose of remimazolam at 0.02–0.05 mg/kg followed by a maintenance dose of 0.20–0.35 mg/(kg·h) could achieve a satisfactory postoperative sedation effect. Our study revealed remimazolam achieved 100% successful short-term sedation at a loading dose of 0.08 mg/kg followed by adjustment rate of 0.1–0.2 mg/(kg·h), with no patients required rescue sedatives in either group. Analysis of titration patterns further revealed similar frequencies of upward and downward adjustments between the LAR and HAR groups, indicating that both adjustment amplitudes afforded comparable titratability without divergent titration pathways to achieve target sedation depth. The observed hemodynamic stability with remimazolam is particularly relevant given that fluid overload and hemodynamic compromise are significant risk factors for ventilator-associated events.^[^^21^^]^ These results highlight remimazolam tosylate’s ability to maintain consistent sedation with minimal dose adjustments, addressing a critical limitation in current ICU sedation strategies.

TEAEs, primarily hypotension, respiratory depression, and bradycardia, aligned with remimazolam’s established safety profile in prior anesthesia studies.^[^^20^^,^^22^^,^^23^^]^ Since sedatives frequently induce blood pressure fluctuation and ICU patients often exhibit hemodynamic instability, the selection of appropriate sedative requires careful consideration.^[^^24^^]^ Remimazolam offers relatively stable hemodynamics and a reduced risk of hypotension.^[^^25^^,^^26^^]^ Tang et al.^[^^12^^]^ reported hypotension occurred in 53.3% patients with remimazolam besylate and 60.0% patients with propofol for deep sedation during short period of mechanical ventilation. In this study under short-term sedation, hypotension occurred in 18.6% patients received remimazolam tosylate. Notably, most TEAEs in our study were mild-to-moderate in severity, with only two patients in HAR group reported unrelated severe TEAEs without treatment discontinuation. The treatment exposure data revealed comparable dosing patterns between groups, with mean durations of approximately 10–11 h for both remimazolam tosylate and concomitant fentanyl administration. These outcomes reinforce remimazolam’s favorable safety margin for ICU patients.

Our PK analysis didn’t show meaningful accumulation with acceptable time to full wakefulness of (6.6 ± 14.3) min, with a mean t_1/2_ of 1.65 h (coefficient of variation [CV]%, 151.6). Notably, despite the observed trend of concentration accumulation and a modest prolongation of t_1/2_ with longer infusion durations, no clinically significant delay in awakening was detected. This dissociation between PK and clinical effects may reflect remimazolam’s rapid clearance via tissue esterase, even under prolonged exposure. Clinically, concentration accumulation appears manageable through infusion rate adjustment under ICU monitoring, without compromising patient safety. Further phase 3 evaluation of pharmacokinetics and accumulation dynamics is warranted.

This study has several limitations. First, the relatively small sample size limits statistical power, highlighting the need for validation in larger phase 3 study. Second, the short-term observation (<24 h) precludes assessment of potential cumulative risks or delirium incidence that might emerge with prolonged administration. Furthermore, the lack of an active comparator group hinders direct comparative analysis, which need to be addressed in future studies to better elucidate the relative advantages of this intervention. Finally, our study population consisted exclusively of postoperative surgical patients, which may limit the generalizability of the findings to medical ICU populations – such as those with sepsis or acute respiratory distress syndrome (ARDS) – who often present with distinct sedation requirements and pharmacodynamic profiles.

## Conclusions

Remimazolam tosylate represents a promising sedative of short-term sedation for mechanically ventilated ICU patients. It provides effective sedation with a manageable safety profile when administered at a loading dose of 0.08 mg/kg, followed by adjustment amplitudes at 0.1 mg/(kg·h) or 0.2 mg/(kg·h). Larger phase 3 trials will further evaluate these benefits.

## Acknowledgements

The authors are grateful to all participants in this trial, including patients, their families and caregivers, the study team and investigators at all the study sites. Medical writing support was provided by Chen Chen, PhD (Jiangsu Hengrui Pharmaceuticals) according to Good Publication Practice Guidelines.

## Funding

This study was funded by Jiangsu Hengrui Pharmaceuticals and partly supported by Guangdong Clinical Research Center for Critical Care Medicine (2020B1111170005).

## Ethics Statement

All procedures were performed in compliance with relevant laws and institutional guidelines and have been approved by the appropriate institutional committees.

## CRediT authorship contribution statement

**Ning Liu:** Writing – review & editing, Writing – original draft, Investigation. **Fenghui Lin:** Writing – review & editing, Validation, Data curation. **Jianli Wen:** Writing – review & editing, Validation, Investigation. **Xiangyou Yu:** Writing – review & editing, Validation, Data curation. **Fachun Zhou:** Writing – review & editing, Investigation. **Zhanbiao Yu:** Writing – review & editing, Investigation. **Mei Yang:** Writing – review & editing, Investigation. **Li Yu:** Writing – review & editing, Investigation. **Ailian Lv:** Writing – review & editing, Investigation. **Yun Sun:** Writing – review & editing, Investigation. **Feng Shen:** Writing – review & editing, Investigation. **Bin He:** Writing – review & editing, Investigation. **Zhenjie Hu:** Writing – review & editing, Investigation. **Tengrui Yin:** Writing – review & editing, Formal analysis. **Xi Li:** Writing – review & editing, Formal analysis. **Huixin Zhao:** Writing – review & editing, Project administration. **Jianfeng Wu:** Writing – review & editing, Investigation. **Chuanxi Chen:** Writing – review & editing, Investigation. **Yao Nie:** Writing – review & editing, Investigation. **Lingyun Zuo:** Writing – review & editing, Investigation. **Xiangdong Guan:** Writing – review & editing, Supervision, Conceptualization. **Minying Chen:** Writing – review & editing, Supervision, Conceptualization.

## Conflict of Interest

Tengrui Yin, Xi Li, Huixin Zhao report employment by Jiangsu Hengrui Pharmaceuticals. All other authors declare that they have no known competing financial interests or personal relationships that could have appeared to influence the work reported in this paper.

Given his role as Editorial Board Member, Jianfeng Wu had no involvement in the peer-review of this article and has no access to information regarding its peer-review. Full responsibility for the editorial process for this article was delegated to another journal editor.

## Appendix Supplementary materials

Image, application 1
