# Supplementary material for: Efficacy and safety of remimazolam tosylate for sedation in ICU patients: A multicenter, randomized, phase 2 study
Source: J Intensive Med. 2026 Mar 28;6(3):247–54. doi: 10.1016/j.jointm.2026.01.009 (PMC13184473; doi:10.1016/j.jointm.2026.01.009)
Supplement: Supplementary file 1 [file mmc1.docx]

Supplementary Materials

## Supple**mentary method**s

**Sedation and analgesia prior to initiation of remimazolam tosylate**

After the patients were enrolled and admitted to ICU, if sedation was needed before initiation of remimazolam tosylate, propofol could be administered as continuous intravenous infusion within 0.3–4.0 mg/kg/h. After randomization, propofol must be discontinued. Following discontinuation, it was recommended to perform Richmond Agitation-Sedation Scale (RASS) assessments every 15 ± 5 min, or as needed by the investigator. If any RASS assessment within 1 hour after discontinuing propofol is ≥ +2, the study drug could be initiated.

After randomization and prior to initiating remimazolam tosylate, if analgesia was needed, only a single intravenous bolus of 0.5 μg/kg or a continuous intravenous infusion at 0.5-1.0 μg/kg/h of fentanyl citrate was permitted.

**CPOT assessment**

CPOT evaluations were performed at 4 min, 30 min, 1 h, 2 h, 4 h, 6 h, 8 h, 12 h, 16 h, 20 h, and 24 h after initiation of remimazolam tosylate. Immediate assessment was conducted upon discontinuation of remimazolam tosylate, after which no further scheduled CPOT evaluations occurred. Pre-procedural CPOT scoring was required within 5 minutes before any fentanyl infusion rate adjustment, with post-adjustment assessment at 5 min. For sufentanil rescue analgesia, CPOT was evaluated within 3 min before and 3 min after administration. Rationale documentation was mandated prior to any fentanyl bolus administration. Duplicate assessments were waived when procedural timepoints coincided with scheduled evaluations. Investigators could perform additional CPOT assessments at their discretion based on clinical need.

**Calculation of Sedation Success**

The primary endpoint, sedation success, was calculated based on prospectively collected Richmond Agitation-Sedation Scale (RASS) scores and precisely timed drug administration records. RASS was assessed at predefined scheduled time points and at unscheduled times related to clinical interventions, such as before and after infusion rate adjustments, additional bolus doses, or rescue medication. The start and stop times of remimazolam tosylate infusion, from loading dose initiation to discontinuation, were recorded to the minute.

The total study drug administration time was defined as the interval from the loading dose start to the maintenance infusion stop, including any infusion pauses (pump rate = 0 mL/h), and was calculated in minutes. The total time within the target sedation level (RASS –2 to +1) was determined by partitioning the entire administration period into consecutive intervals based on the actual sequence of all RASS assessments. The duration attributable to each RASS score was defined as the time interval from that assessment to the next subsequent assessment. The total time within the target range was the sum of all intervals where the RASS score was between –2 and +1.

A predefined statistical analysis plan was followed to handle missing RASS scores. Missing scores at planned time points were primarily imputed using linear interpolation based on the two adjacent non-missing scores. For other scenarios: scores missing before the first non-missing value were imputed with the median score from the same treatment group; scores missing after the last non-missing value were imputed using the last observation carried forward (LOCF) method. Furthermore, RASS scores documented during periods of rescue sedation or protocol-prohibited infusion rates were replaced using the LOCF method (the last score before the event).

For each patient, the percentage of time within the target sedation level was calculated as: (Total time within target RASS [min] / Total study drug administration time [min]) × 100%. A patient was classified as a "sedation success" only if this percentage was ≥70% and no rescue sedative (propofol) was administered during the remimazolam infusion.

Figure S1. Study design


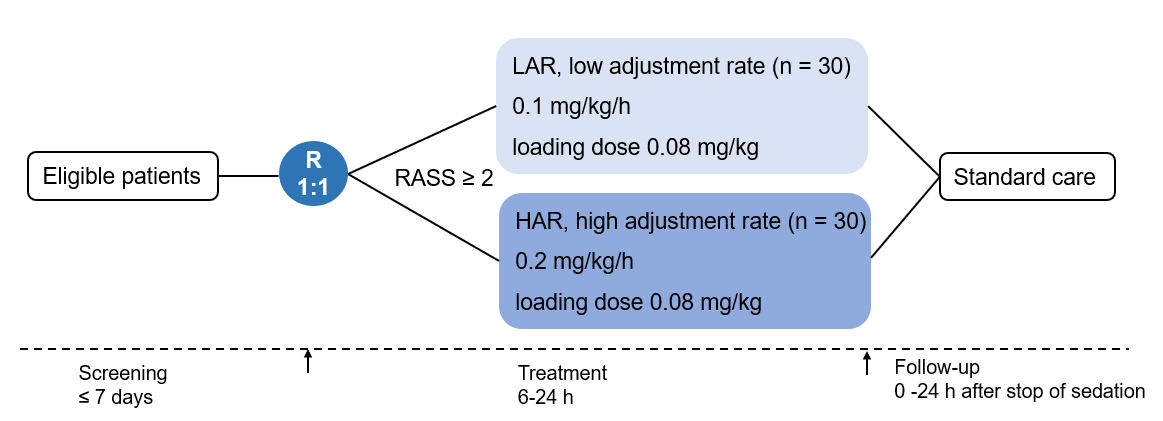


Figure S2. Kaplan-Meier curve of duration of mechanical ventilation


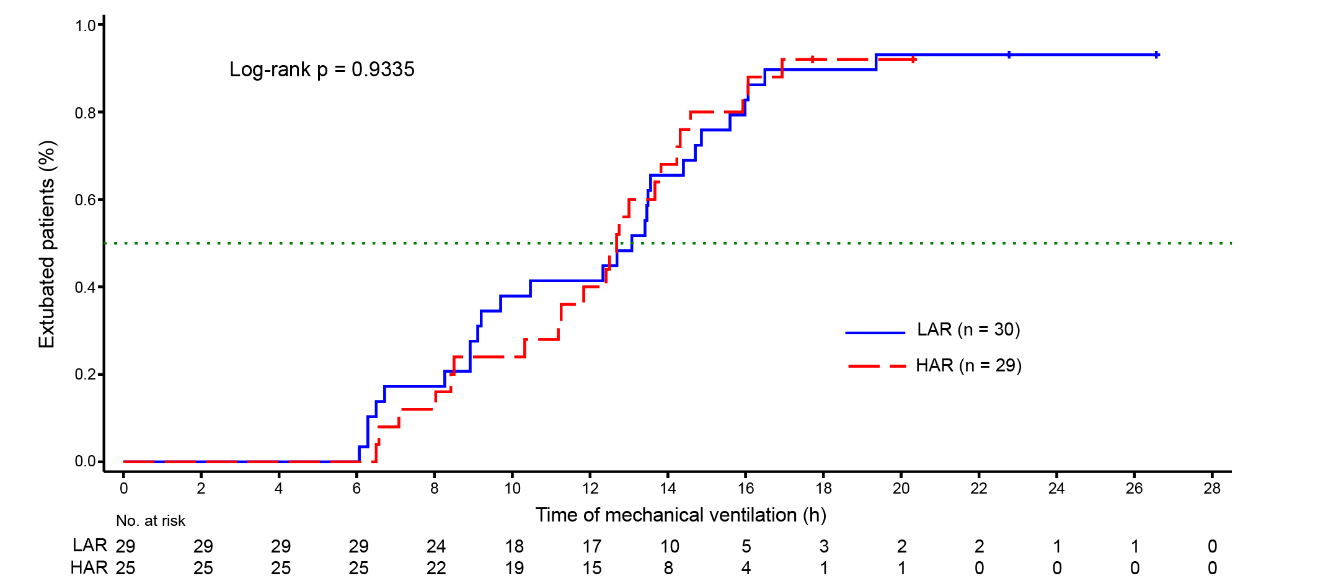


Figure S3. Kaplan-Meier curve of time to full wakefulness.


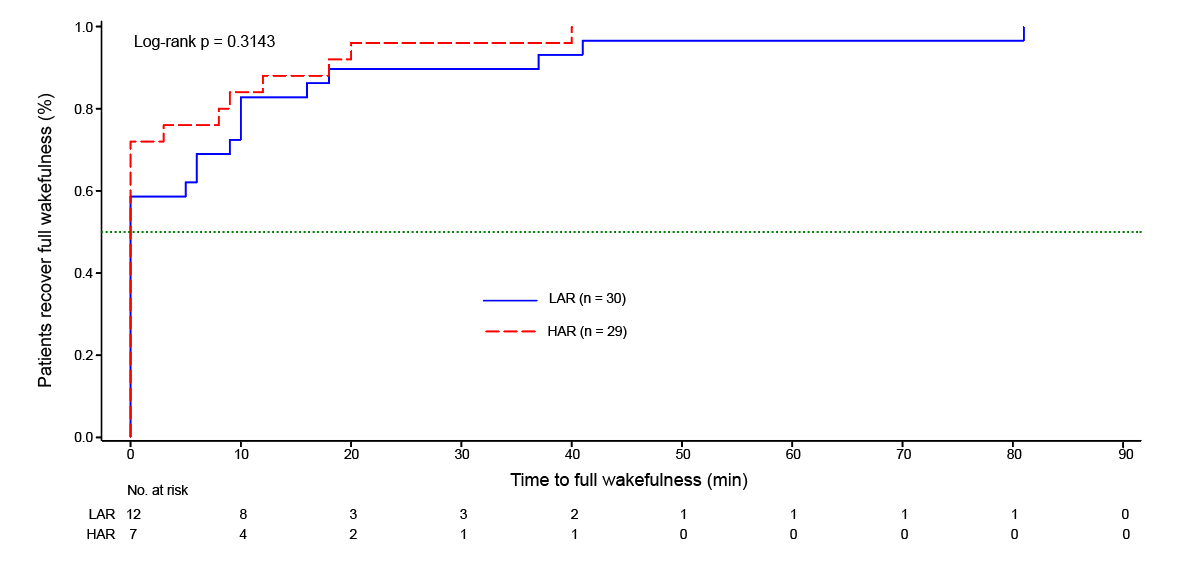


Table S1. List of investigators and participating sites

| **Principal**  **investigator** | **Sites** | **No. of patients enrolled** |
| --- | --- | --- |
| Fenghui Lin | Fujian Provincial Hospital | 14 |
| Xiangdong Guan | The First Affiated Hospital of Sun Yat-sen University | 8 |
| Jianli Wen | The First People's Hospital of Zunyi City | 6 |
| Xiangyou Yu | The First Affiliated Hospital of Xinjiang Medical University | 4 |
| Fachun Zhou | The First Affiliated Hospital of Chongqing Medical University | 4 |
| Zhanbiao Yu | Affiliated Hospital of Hebei University | 4 |
| Mei Yang | The Qujing No.1 People's Hospital | 4 |
| Li Yu | Wuhan Central Hospital | 3 |
| Ailian Lv | The First Hospital of Changsha City | 3 |
| Yun Sun | The Second Hospital of Anhui Medical University | 2 |
| Feng Shen | The Affliliated Hospital of Guizhou Medical University | 2 |
| Bin He | Shanghai Chest Hospital | 2 |
| Zhenjie Hu | The Fourth Hospital of Hebei Medical University | 1 |

Table S2. Inclusion/exclusion criteria

| **Inclusion criteria** |
| --- |
| 1. Patients or their guardians able to provide written informed consent and agrees to comply with study requirements and procedures. 2. Currently receiving mechanical ventilation via endotracheal intubation, with an anticipated need for continuous sedation for at least 6 h after randomization, targeting a Richmond Agitation-Sedation Scale (RASS) score between -2 and +1. 3. Aged 18 to 80 years, any sex. 4. Body mass index (BMI) >18 kg/m^2^ to <30 kg/m^2^. |
| **Exclusion Criteria** |
| 1. Conditions Requiring Deep Sedation in ICU:   a) Severe ventilator dyssynchrony;  b) Severe traumatic brain injury with intracranial hypertension;  c) Severe Acute Respiratory Distress Syndrome (ARDS).   1. Anticipated need for neuromuscular blocking agents during sedation (excluding intubation). 2. Blindness, deafness, or other conditions significantly impairing RASS assessment. 3. History of psychiatric disorders (e.g., schizophrenia, major depression) or cognitive dysfunction. 4. Sequential Organ Failure Assessment (SOFA) score >9 prior to randomization. 5. Receiving dialysis at screening or anticipated to require dialysis during the study period. 6. History of epilepsy or status epilepticus at screening. 7. History of abuse of psychotropic or anesthetic agents. 8. Current diagnosis or history of myasthenia gravis. 9. Bradycardia requiring pharmacological treatment at screening (excluding intraoperative events), or history of severe arrhythmias (e.g., Type II second-degree or higher atrioventricular block), excluding subjects with pacemakers. 10. History of myocardial infarction or unstable angina within 6 months prior to randomization. 11. Post-neurosurgical procedure (e.g., craniotomy, spinal surgery), neurological dysfunction, and/or coma. 12. Conditions not requiring continuous sedation during mechanical ventilation (e.g., Guillain-Barré syndrome). 13. Requirement for vasoactive agents to maintain blood pressure at screening (excluding intraoperative use only). 14. Laboratory Abnormalities:   a) Hematology: Hemoglobin (Hb) ≤70 g/L OR Platelet count (PLT) ≤50 × 10⁹/L.  b) Liver Function: Alanine aminotransferase (ALT) ≥3 × Upper Limit of Normal (ULN) AND Total Bilirubin (TBiL) ≥2 × ULN.  c) Renal Function: Serum Creatinine (Cr) ≥1.5 × ULN.   1. Coagulopathy (Pre-operative): Prothrombin Time (PT) ≥1.5 × ULN, or Activated Partial Thromboplastin Time (APTT) ≥1.5 × ULN. 2. Known hypersensitivity to benzodiazepines, propofol, fentanyl, sufentanil, or any excipients of these drugs. 3. Pregnant or breastfeeding women. 4. Plans for pregnancy within 28 days after the trial, or unwillingness/inability to use protocol-specified contraceptive methods. 5. Participation in any other investigational drug or device clinical trial within 3 months prior to randomization (defined as receipt of investigational product/device or placebo). 6. Any other condition deemed by the Investigator to make the subject unsuitable for study participation. |

Table S3. Titration direction analysis

|  | LAR group  (n = 30) | HAR group  (n = 29) | P value |
| --- | --- | --- | --- |
| **Titration direction** |  |  |  |
| Number of upward adjustments |  |  |  |
| Mean ± SD | 1.3 ± 1.8 | 1.1 ± 1.8 |  |
| Median (IQR) | 0.5 (0-3.0) | 0 (0-1.0) | 0.6638 |
| Number of downward adjustments |  |  |  |
| Mean ± SD | 1.3 ± 1.7 | 1.3 ± 1.9 |  |
| Median (IQR) | 1.0 (0-2.0) | 0 (0-2.0) | 0.7026 |

LAR, low adjustment rate; HAR, high adjustment rate.

The comparisons were performed using the Wilcoxon rank-sum test.

Table S4. Treatment exposure of remimazolam tosylate

|  | LAR group  (n = 30) | HAR group  (n = 29) |
| --- | --- | --- |
| Duration of administration, min |  |  |
| Mean ± SD | 624.1 ± 196.6 | 664.7 ± 267.3 |
| Range | 360.0 - 1113.7 | 360.0 - 1437.0 |
| Loading dose (mean ± SD), mg | 4.9 ± 0.9 | 5.1 ± 0.9 |
| Total dose (mean ± SD), mg | 138.5 ± 101.7 | 143.7 ± 88.1 |
| Additional dose, *n* (%) |  |  |
| Yes | 1 (3.3) | 2 (6.9) |
| No | 29 (96.7) | 27 (93.1) |
| Total additional dose (mean ± SD), mg | 4.1 ± NA | 4.9 ± 1.0 |
| Intravenous infusion rate (mean ± SD), mg/kg∙h | 0.21 ± 0.10 | 0.20 ± 0.08 |

LAR, low adjustment rate; HAR, high adjustment rate.

Duration: time from drug loading to discontinuation.

Table S5. Pharmacokinetics parameters of remimazolam tosylate (N=26)

|  | HR7056 | HR7054 |
| --- | --- | --- |
| C_max_, mg/L | 266.5 (72.6) | 2967.3 (80.1) |
| AUC_0-t_, h·μg/mL | 2344.8 (86.8) | 28225.1 (76.9) |
| t_1/2_, h | 1.65 (151.6) | 3.67 (74.7) |
| CL/F, L/h | 50.0 (49.5) | - |
| V_z_/F, L | 119.1 (42.5) | - |

Data are mean (CV%) for t_1/2_; and GeoMean (CV%) for other parameters.

C_max_, maximum plasma concentration; AUC_0-t_, area under the concentration-time curve from zero to the last measurable concentration; t_1/2_, elimination half-life; CL, apparent clearance; V_z_, apparent volume of distribution.
